# Analysis of the Murine Immune Response to Pulmonary Delivery of Precisely Fabricated Nano- and Microscale Particles

**DOI:** 10.1371/journal.pone.0062115

**Published:** 2013-04-12

**Authors:** Reid A. Roberts, Tammy Shen, Irving C. Allen, Warefta Hasan, Joseph M. DeSimone, Jenny P. Y. Ting

**Affiliations:** 1 Department of Microbiology and Immunology, University of North Carolina, Chapel Hill, North Carolina, United States of America; 2 Eshelman School of Pharmacy, University of North Carolina, Chapel Hill, North Carolina, United States of America; 3 Department of Biomedical Sciences and Pathobiology, Virginia-Maryland Regional College of Veterinary Medicine, Virginia Polytechnic Institute and State University, Blacksburg, Virginia, United States of America; 4 Department of Chemistry, University of North Carolina, Chapel Hill, North Carolina, United States of America; 5 Lineberger Comprehensive Cancer Center, University of North Carolina, Chapel Hill, North Carolina, United States of America; 6 Department of Biochemistry and Biophysics, University of North Carolina, Chapel Hill, North Carolina, United States of America; 7 Carolina Center of Cancer Nanotechnology Excellence, University of North Carolina, Chapel Hill, North Carolina, United States of America; 8 Department of Chemical and Biomolecular Engineering, North Carolina State University, Raleigh, North Carolina, United States of America; 9 Department of Pharmacology, University of North Carolina, Chapel Hill, North Carolina, United States of America; 10 Institute for Advanced Materials, University of North Carolina, Chapel Hill, North Carolina, United States of America; 11 Institute for Nanomedicine, University of North Carolina, Chapel Hill, North Carolina, United States of America; 12 Sloan-Kettering Institute for Cancer Research, Memorial Sloan-Kettering Cancer Center, New York, New York, United States of America; National Institute of Health (NIH), United States of America

## Abstract

Nanomedicine has the potential to transform clinical care in the 21^st^ century. However, a precise understanding of how nanomaterial design parameters such as size, shape and composition affect the mammalian immune system is a prerequisite for the realization of nanomedicine's translational promise. Herein, we make use of the recently developed Particle Replication in Non-wetting Template (PRINT) fabrication process to precisely fabricate particles across and the nano- and micro-scale with defined shapes and compositions to address the role of particle design parameters on the murine innate immune response in both *in vitro* and *in vivo* settings. We find that particles composed of either the biodegradable polymer poly(lactic-co-glycolic acid) (PLGA) or the biocompatible polymer polyethylene glycol (PEG) do not cause release of pro-inflammatory cytokines nor inflammasome activation in bone marrow-derived macrophages. When instilled into the lungs of mice, particle composition and size can augment the number and type of innate immune cells recruited to the lungs without triggering inflammatory responses as assayed by cytokine release and histopathology. Smaller particles (80×320 nm) are more readily taken up *in vivo* by monocytes and macrophages than larger particles (6 µm diameter), yet particles of all tested sizes remained in the lungs for up to 7 days without clearance or triggering of host immunity. These results suggest rational design of nanoparticle physical parameters can be used for sustained and localized delivery of therapeutics to the lungs.

## Introduction

The application of nanoparticles in medicine for disease treatment is a potentially transformative area of research. The possibility of potent instruction and modulation of host physiology through nanomaterials has been abundantly demonstrated. These efforts include modulation of cell-specific gene expression through delivery of antisense oligonucleotides, dose-sparing and targeted delivery of pharmacologics, as well as enhanced multi-functional imaging diagnostics through nanoformulations [1]–[3]. Another area where nanotechnology may revolutionize clinical care is the ability to direct immune responses in defined manners. The most obvious benefit is in the design of next-generation vaccines against microbial pathogens, whereby antigen specific immune responses can be elicited at levels far more potent than existing vaccines, commensurate with the immune response engendered by live organisms [4]–[6]. These latter advances owe much to our recent understanding of the critical role of innate immunity in contextualizing an appropriate adaptive immune response. This context is triggered through endogenous host receptor signaling pathways, most well characterized by the TLR family of pattern recognition receptors (PRR), but hallmarked by a panoply of such PRRs including C-type lectin receptors, RIG-like helicases and the burgeoning understanding of Nod-like receptors (NLRs) as sentinels of the intracellular environment [7].

Researchers in the emerging field of immune-engineering are capitalizing on these exciting advances to open up the possibility to direct and instruct immunological outcomes to a variety of pathological conditions [8]. These include improving current pathogen vaccines, to the more nascent fields of cancer immunotherapy, tolerance induction in the setting of autoimmunity and organ transplantation, as well as general immunological rebalancing in diseased settings, such as the chronic inflammation associated with type 2 diabetes. The implications of such technology are profound and potentially represent a paradigm shift in clinical practice across a broad swath of medicine. However, efforts to use nanotechnology and material sciences engineering to modulate human biology *in situ* require a comprehensive understanding of the immune response, or lack thereof, engendered to introduced nanocarriers [9], [10].

In order for the gamut of potential downstream therapeutic applications of nanomedicine to be realized, we must first understand how the physical properties of nanomaterials augment host immune responses. These principles will then enable the appropriate design of nano- and micro-scale interventions for specific purposes. For example, the use of nanoparticles to deliver potent biological molecules, such as oligonucleotides or small molecules augmenting intracellular signaling pathways, may squander the regulatory opportunity to reach the clinic if the nanocarriers for such entities initiate off-target events that activate host immune responses. Conversely, vaccines can be made more potent if nanocarriers are designed to activate the appropriate innate immune response to tailor adaptive immune responses to delivered antigens. As an example, the current state of the art is to use Alum as a non-specific immunomodulatory adjuvant in vaccine formulations. This could explain, in part, why some of the most pressing pathogens do not yet have useful vaccines because Alum is known to engender a mixed Th1/Th2-biased humoral immune response that does not reflect the immune activation which occurs during an actual microbial infection [11]. It is conceivable that nano-carriers designed to elicit the appropriate adaptive immune response to an antigen of interest-i.e., Th17 against fungal pathogens-may enable the generation of vaccine-induced immune responses that more closely mimic the natural immune response elicited by infection with a given pathogen as opposed the non-tailored immunity induced by Alum.

A great issue in advancing nanotechnology from a laboratory pursuit into a component of clinical care is a robust understanding of how physical particle properties augment biological outcomes. There is a wealth of literature promoting the use of nanotechnology in modern medicine, but much of this literature relies on particle fabrication methods, such as oil-in-water emulsion, that generate heterogeneous populations of particles that can vary widely between batches and across labs [12], [13]. In addition, most studies related to biomedical applications of nanotechnology have not addressed the role of the immune system in the host response to particulate delivery, a critical issue that threatens to diminish the utility of such particles if these are rapidly cleared by innate immune cells and/or induce localized or systemic immune responses that pose unintended complications for clinical development. In cases where the immunological parameters of nanotechnology are being addressed, great variance is seen based on size, composition and even surface modification of particles, highlighting the tremendous complexity and exquisite sensitivity of the immune system to nanoscale events [9], [10], [14].

Most currently employed fabrication methods do not allow precise control over particle physical parameters, thus it is difficult to draw conclusions as to how size, shape and composition affect the innate immune response to particles in the nano and micron range. To address this lack of knowledge, we employed our recently developed top-down nanofabrication technique termed Particle Replication in Non-Wetting Templates (PRINT) [15]–[18]. Using soft-lithography techniques adopted from the semi-conductor industry, PRINT enables the production of monodisperse nano- and microparticles with well-defined control over particle size, shape, composition, modulus and surface chemistry. Therefore, the role of these physical parameters in augmenting biological responses can be reproducibly probed using PRINT technology.

To lend both clinical and field relevance to our findings, particles were designed with either the F.D.A approved polymer Poly-lactic co-gloycolic acid (PLGA) or derivatives of the commonly published hydrogel polymer polyethylene glycol (PEG). While PLGA is an attractive polymer given its long history of clinical use, our study was aimed in part to clarify discrepancy in the literature as to whether particles made of PLGA trigger inflammation. As our main purpose was to define the ‘baseline’ status of whether particles of defined size, shape and composition triggered inflammation, we did not augment particles in this study to include additional biologically active molecules, such as oligonucleotides, small molecules or adjuvants as previously published by our group and others [19]–[22]. To this end, we used *in vitro* assays with murine derived macrophages and *in vivo* delivery of particles to the lungs of mice to test the inflammatory potential of these particles. The lung is a highly desired site for therapeutic delivery of nanomedicine and we chose it for both clinical relevance and its sensitivity as an immunological organ [23]. Our findings imply that the delivery of PRINT nano- and micro-particles do not engender systemic or localized inflammatory responses and may not be impeded by host immune responses to the polymers used in this study. Future design strategies for the panoply of therapeutic opportunities made available by nanoenginneering are likely available, as particles across broad size ranges can thus be rationally designed from an inert state.

## Materials and Methods

### Particle Materials

Poly(ethylene glycol) diacrylate (M_n_ 700) (PEG_700_DA), 2-aminoetheyl methacrylate hydrochloride (AEM), Diphenyl (2,4,6-trimethylbenzoyl)-phoshine oxide (TPO), and poly lactic co-glycolic acid (PLGA; 85∶15 lactic acid/glycolic acid, MW = 55 000 g/mol) were purchased from Sigma-Aldrich. Tetraethylene glycol monoacrylate (HP4A) was synthesized in-house as previously described [24]. Thermo Scientific Dylight 650 maleimide, PTFE syringe filters (13 mm membrane, 0.220 µm pore size), dimethylformamide (DMF), triethanolamine (TEA), pyridine, sterile water, borate buffer (pH 8.6), Dulbecco's phosphate buffered saline (DPBS) (pH 7.4), 1X phosphate buffered saline (PBS) (pH 7.4), acetic anhydride and methanol were obtained from Fisher Scientific. Conventional filters (2 µm) were purchased from Agilent and polyvinyl alcohol (Mw 2000) (PVOH) was purchased from Acros Organics. All PRINT molds used in these studies (80 nm×320 nm, 1 µm cylinder, 1.5 µm and 6 µm donuts) were kindly provided by Liquidia Technologies.

### PRINT PLGA Particle Fabrication

The PRINT process for fabricating particles has been described previously [21], [22]. Briefly, to fabricate PLGA particles, a preparticle solution containing PLGA was prepared in a DMSO/DMF/water solvent mixture (4∶16∶1) and cast on a poly-(ethylene teraphthalate) (PET) sheet (delivery sheet) using a #5 Mayer Rod (R.D. Specialties). The delivery sheet was placed in contact with a PRINT mold with desired features patterned (e.g., 80×320 nm). The delivery sheet and mold were passed through a heated laminator (150°C, 5.5×105 Pa) and separated at the nip. This heating process enables the PLGA polymer solution to fill the molds, thereby forming nanoparticles of desired size and shape. Nanoparticles were then harvested from the PRINT mold by placing it in contact with a PET sheet coated with a layer (400 nm cast from water) of poly(vinyl alcohol) (PVA, MW = 2000 g/mol). This mold/PET-PVA ensemble was then passed through the laminator (150°C, 5.5×105 Pa) to transfer the nanoparticles to the PVA sheet. Both laminator steps, the filling of the mold and transfer of particles onto the PVA-coated PET sheet, were performed at low humidity (∼20–30%). Particles were released from the PET/PVA sheet by delivering ∼1 ml of sterile water via a bead harvester to dissolve the PVA layer and remove the particles from the PET sheet. A typical yield of 80×320 nm PLGA particles was ∼0.4 mg particles/ft of PRINT mold, though this depended on the particle feature size of the mold. To remove excess PVA and concentrate the particles, tangential flow filtration (TFF; Spectrum Labs) was used to concentrate particles in sterile water (1–2 mg/ml). For later use in particle characterization assays and experiments, particles were lyophilized by adding 10× mannitol and 8× sucrose (10× and 8× to mass of particles) using a tree lyophilizer. Mannitol and sucrose were used as cryoprotectants.

### PRINT Hydrogel Fabrication

The process of fabricating 80×320 nm hydrogel particles was conceptually similar to PLGA fabrication, but with important differences. The pre-particle solution (PPS) contained a composition of 67.5 wt% HP_4_A, 20 wt% AEM (functional monomer), 10 wt% PEG_700_DA (crosslinker), 1 wt% TPO (photo initiator) and 1.5 wt% Dylight 650 maleimide. This composition was then dissolved at 3.5 wt% in methanol and drawn as a thin film using a # 3 Mayer rod (R.D. Specialties) onto a roll of corona treated PET using an in-house custom-made roll-to-roll lab line (Liquidia Technologies) running at 12 ft/min. The solvent was evaporated from this delivery sheet by exposing the film to heat guns. The delivery sheet was laminated (80 PSI, 12 ft/min) to the patterned side of the mold, followed by delamination at the nip. Particles were cured by passing the filled mold through a UV-LED (Phoseon, 395 nm, 3 SCFM N_2_, 12 ft/min). A PVOH harvesting sheet was hot laminated to the filled mold (140°C, 80 PSI, 12 ft/min). Upon cooling to room temperature, particles were removed from the mold by splitting the PVOH harvesting sheet from the mold. Particles were then harvested by dissolving the PVOH in a bead of water (1 mL of water per 5 ft of harvesting sheet). The particle suspension was passed through a 2 µm filter (Agilent) to remove any large particulates. To remove the excess PVOH, particles were centrifuged (Eppendorf Centrifuge 5417R) at 14000 rpm for 15 min, the supernatant was removed and the particles were re-suspended in sterile water. This purification process was repeated 4 times prior to lyophilization as detailed above. The 80×320 nm particles were acetylated prior to experimental use to match negative charge of micron sized hydrogel particles.

The 1.5 and 6 µm donut shaped hydrogel particles were fabricated using a dropcast method. The pre-particle solution (PPS) was composed of 20% PEG_700_-DA, 78% HP_4_A, 1% TPO (photoinitiator), and 1% Dylight 650. The solution was spread onto a fluorocur mold and a poly(ethylene terephthalate) (PET) sheet was laminated on top of the mold and polymer mixture and run through a heated, pressurized laminator to fill the molds. The mold was then cured with a UV LED lamp for 30 seconds. Particles were transferred out of the mold onto a Luvitec harvesting layer by laminating the mold and Luvitec sheet together and running them through a heated laminator nip. The mold and harvesting sheet was separated, leaving free particles on the harvest layer. Particles were collected from the harvest sheet by bead harvesting with water and pelleted by centrifugation. The particles were re-suspended in tert-butanol and lyophilized overnight.

### Particle Characterization

Thermogravimetric analysis (TGA) was used to determine stock particle concentrations (TA Instruments Q5000 TGA). Briefly, 20 µL of the stock nanoparticle solution was pipetted into a tared aluminum sample pan. The sample was heated at 30°C/min to 130°C and held at this temperature for 10 minutes. The sample was then cooled at 30°C/min down to 30°C and held for 2 minutes. A Hitachi S-4700 scanning electron microscope (SEM)was used to visualize particles. Prior to imaging, the SEM samples were coated with 1.5 nm of gold-palladium alloy using a Cressington 108 auto sputter coater. Particle size and zeta potential were measured by dynamic light scattering (DLS) on a Zetasizer Nano ZS (Malvern Instruments, Ltd.).

### Experimental animals

All studies were conducted in accordance with National Institutes of Health guidelines for the care and use of laboratory animals and approved by the Institutional Animal Care and Use Committee (IACUC) of the University of North Carolina at Chapel Hill. All animals were maintained in pathogen-free facilities at the University of North Carolina at Chapel Hill.

### In vitro confocal analysis of hydrogel particle uptake

MH-S murine alveolar macrophages were plated in complete DMEM at 20,000 cells per well in 8-well chamber slides (LabTek) 48 hours prior to treatment with particles. Particles were resuspended in DMEM at 20 µg/ml and 300 µl of particle solution were added to each well. Particles were incubated with cells at 37°C for 4 hours. Cells were then washed twice with PBS and cells fixed with 4% Paraformaldehyde (PFA) solution and later stained with Alexa Fluor 488 Phalloidin (Invitrogen) and DAPI (Vectashield, Vector Labs). Fluorescent imaging of stained cells was performed on a Zeiss 710 laser scanning confocal imaging system (Zeiss).

### In vitro inflammation assays

Bone marrow macrophages were isolated from the femurs of C57Bl/6 and BALB/c mice using standard procedures. Bone marrow-derived macrophages were cultured for six days in DMEM supplemented with 10% fetal bovine serum, L-Glutamine, pen/strep and 20% L929-conditioned medium prior to use in particle experiments. Adherent cells were isolated and plated in complete Dulbecco's Modifed Eagle Medium (Gibco) with 10% fetal calf serum, 1% penicillin/streptomycin and 1% L-glutamine at 200,000 cells per well in a 96-well dish for 24 hours prior to treatment with particles for up to 24 hours. Some cells were primed with LPS (50 ng/ml) for 24 prior to particle treatment to provide signal 1 for inflammasome activation. Monosodium urate (MSU; Invivogen) treated at 300 µg/ml or ATP (5 mM; Sigma-Aldrich) was used as a positive control for inflammasome activation. Particles were resuspended in PBS prior to dosing at various concentrations. After 24 hours of particle treatment in triplicate, supernatants were harvested and analyzed by murine IL-1β, TNF-α and IL-6 ELISA (BD Biosciences). Limit of detection for ELISAs was 31.3 pg/ml (IL-1β) and 15.6 pg/ml (TNF-α and IL-6). Lactate dehydrogenase release was used to measure particle-induced cytotoxicity (Roche Applied Sciences).

### Assessment of airway inflammation

To assess whether PRINT particles induce airway inflammation, 10–12 week old female C57Bl/6 mice were anesthetized with isofluorane inhalation and particles were instilled via intratracheal (i.t.) administration. 50 µg of particles were dosed in 50 µl of PBS. Intratracheal administration of PBS (50 µl) or LPS (20 µg in 50 µl PBS) served as negative and positive controls for airway inflammation, respectively, as previously described [25]. Mice were euthanized and airway inflammation was assessed 48 hours or 7 days post treatment.

Serum was collected from animals by cardiac puncture and centrifuged at 15,000 RPM for 10 minutes. The serum supernatant was collected and used for ELISA analysis of inflammatory markers. Bronchoalveolar lavage fluid (BALF) was also collected to evaluate local leukocyte and cytokine levels in the lungs. For this purpose, lungs were lavaged three times with 1 ml Hanks Balanced Salt Solution (HBSS; Gibco). After centrifugation at 1500 RPM for 5 minutes, cell-free supernatants were collected and used to assess cytokine levels of IL-1β, TNF-α and IL-6 via ELISA (BD Biosciences). RBC were lysed via brief hypotonic saline treatment and the cell pellet was resuspended in PBS. Total BALF cellularity was assessed with a hemacytometer. The cellular composition was determined by cytospin of BALF aliquots onto slides and staining with Diff-Quik (Dade Behring) for differential cell counts. Leukocytes were identified based on the morphology of ≥200 cells per sample. Following BALF harvest, the lungs were fixed by inflation (20-cm pressure) and immersed in 10% buffered formalin.

### Histopathological examination

Inflammation was evaluated in 5 µm sections of the left lung lobe after hematoxylin and eosin (H&E) staining. Serial paraffin-embedded sections were set and cut to reveal maximum longitudinal visualization of the intrapulmonary main axial airway and inflammation was scored by one of the authors (I.C.A.) who was blinded to genotype and treatment. As previously described, histology images were evaluated on each of the following inflammatory parameters and scored between 0 (absent) to 3 (severe): mononuclear cell infiltration, polymorphonuclear cell infiltration, airway epithelial cell hyperplasia/injury, extravasation, perivascular cuffing, and estimated percentage of the lung involved with inflammation [26], [27]. Scores for each parameter were averaged for a total histology score.

### Particle uptake in BALF

BALF aliquots from PEG treated mice were fixed in 2% paraformaldehyde and stained with DAPI (nuclei) and Phalloidin 488 (actin) and then viewed via epifluorescence microscopy for particle uptake (Dylight 650). Five distinct fields of view (FOV) were captured for each slide. The percentage of cell uptake was determined by dividing the number of cells showing particle internalization by the total number of cells in each field of view.

### Statistical Analysis

GraphPad Prism 5 software was used to identify statistical significance. Single data point comparisons were evaluated by Student's two-tailed t-test, whereas multiple comparisons were evaluated for statistical significance using Analysis of Variance (ANOVA) followed by Tukey-Kramer HSD post-test. All cytokine and cell count data are presented as mean +/− standard deviation (SD) or standard error of the mean (SEM), respectively, with a p-value less than 0.05 considered statistically significant.

## Results

### PRINT enables the fabrication of monodisperse and homogenous particles

We employed Particle Replication in Non-Wetting Templates (PRINT) in an effort to address whether particles of defined size, shape and composition trigger an inflammatory response in mice. This fabrication platform enables production of homogenous and monodisperse particles with user-defined physical parameters. As a large amount of literature shows crucial biological differences depending on size and shape, the PRINT technique enables reproducible probing of basic cell biology with nearly complete control of design parameters [10], [28]–[30].

**Figure 1:**
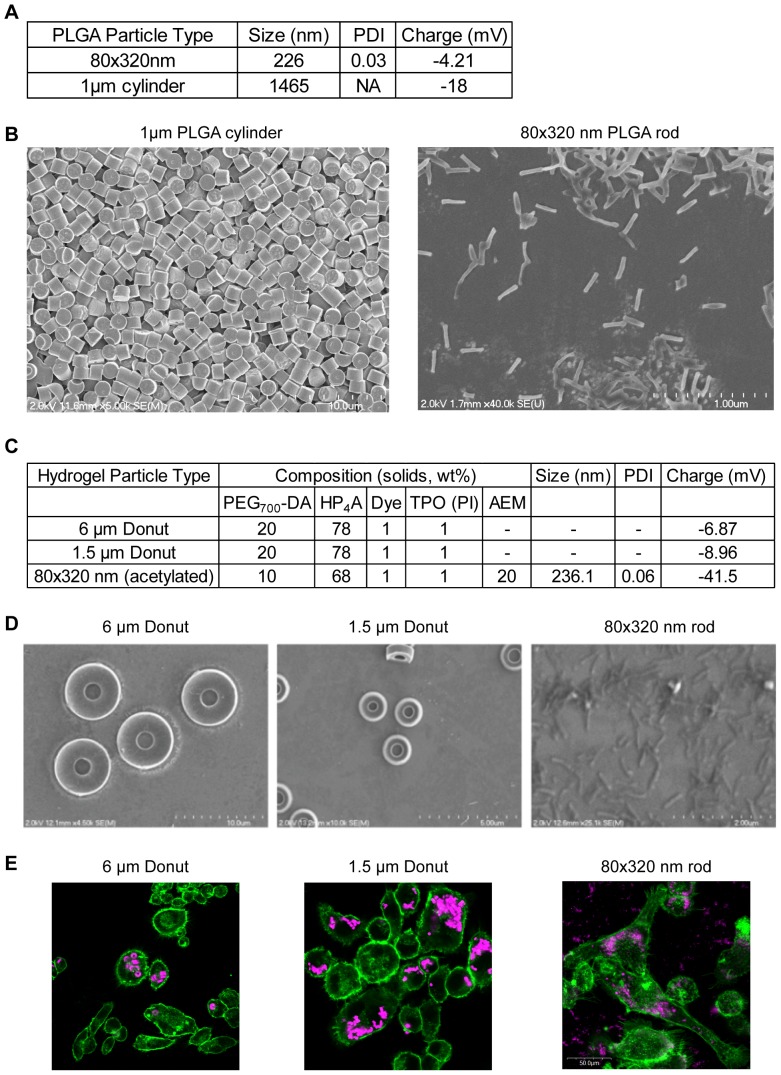
PRINT Particle Characterization. **A)** Dynamic light scattering (DLS) and zeta potential measurements of PLGA particles used in studies. Particle charge decreases with increasing size. **B)** Scanning electron microscope (SEM) images of PLGA particles. **C)** PEG particle composition and characterization. **D)** SEM of PEG particles. **E)** Confocal images of hydrogel particle uptake in MH-S alveolar macrophage cells after 4 hours of treatment. Scale bar is 50 µm.

For the purposes of our studies, we fabricated particles across the nano and micron range to reflect biologically relevant sizes. These include 80×320 nm particles (commensurate with the sizes of small bacteria and large viruses), 1 µm and 1.5 µm particles (commensurate with bacteria and platelets), and 6 µm particles (akin to a red blood cell in size) [28], [31]. To characterize the fabricated particles, we performed dynamic light scattering (DLS) and zeta potential measurements as shown in Figure 1A. Note that DLS measurements are quantified based on particles with a perfect sphere shape, so that the size ranges detected are in line with non-spherous shapes of the molds used. The poly-dispersity index (PDI), a measure of heterogeneity in a particle population, indicates we were able to fabricate monodisperse particles of the same size and shape. This is further evidenced by scanning electron microscopy images (Figure 1B). As the surface charge of particles has been shown to play a role in biological outcomes, such as protein adsorption and cell uptake, we measured the Zeta potential of particles to quantify net surface charge [30], [32], [33]. For all PLGA particles, surface charge was negative and decreased with increasing particle size (Figure 1A).

While our initial studies used particles fabricated from the F.D.A-approved biocompatible and biodegradable polymer *poly(lactic-co-glycolic acid*) PLGA, we also incorporated studies using hydrogel particles fabricated with derivatives of biocompatible poly (ethylene glycol) (PEG). Chemical modification of PEG is more feasible than with PLGA and thus it is often used to add increased functionality to nanocarriers, such as decoration of cell-targeting ligands, imaging agents, and pharmacologic cargo incorporation. Characteristics of fabricated PEG particles were similar to that of the PLGA particles, with low PDI and negative surface charge (Figure 1C) and monodisperisty as evidenced by SEM (Figure 1D). These PEG particles were fabricated with a dye (DyLight 650) to enable fluorescent imaging of particles in downstream assays and may also be referred to as hydrogels hereafter. To validate hydrogel particle uptake in a pertinent pulmonary immune cell population, we performed confocal analysis using the MH-S murine alveolar macrophage cell line. As shown in Figure 1E, all particle sizes are taken by four hours after treatment.

### PLGA particles do not induce inflammation by bone marrow-derived macrophages

Much work has been done *in vitro* to assess the potential use of PLGA nanoparticles in a variety of therapeutic modalities, from delivery of chemotherapeutics and siRNA to imaging agents for improved diagnostics. However, less work has been done to characterize the innate immune response to such particles and whether physical parameters of particles can augment the immune response. As a primary sentinel of host homeostasis, the innate immune system is tasked with identifying foreign matter in the body and initiating an appropriate response. Subsequent activation of the innate immune response is hallmarked by release of soluble protein messengers like cytokines that serve to recruit other immune cells to the area to participate in defense and repair of the host [7]. This inflammatory response is initiated by release of pro-inflammatory cytokines, including TNF-α, IL-6 and IL-1β, from innate immune cells, such as macrophages.

The field of environmental toxicology has long studied the role of nanoparticulates in inducing inflammation, in particular in the lung [34]. Attempting to synthesize work by other groups using a range of particle compositions and sizes suggest that there is no clear correlation between the physical parameters of a particle and the ensuing inflammatory response to it. Generally speaking, the composition of a particle has greater bearing on the inflammatory response than its size or shape. As an example, titanium dioxide and silica dioxide nanoparticles trigger inflammation, whereas zinc oxide nanoparticles do not, even though all particles were of similar size (15–20 nm) [35]. Others have identified size-dependent inflammation and cell death that could be inhibited simply with surface modification of silica particles with common chemical groups such as aldehydes [36]–[38]. These findings highlight the sensitivity of the innate immune system as each particle may engender unique responses depending on its size, shape and composition.

**Figure 2:**
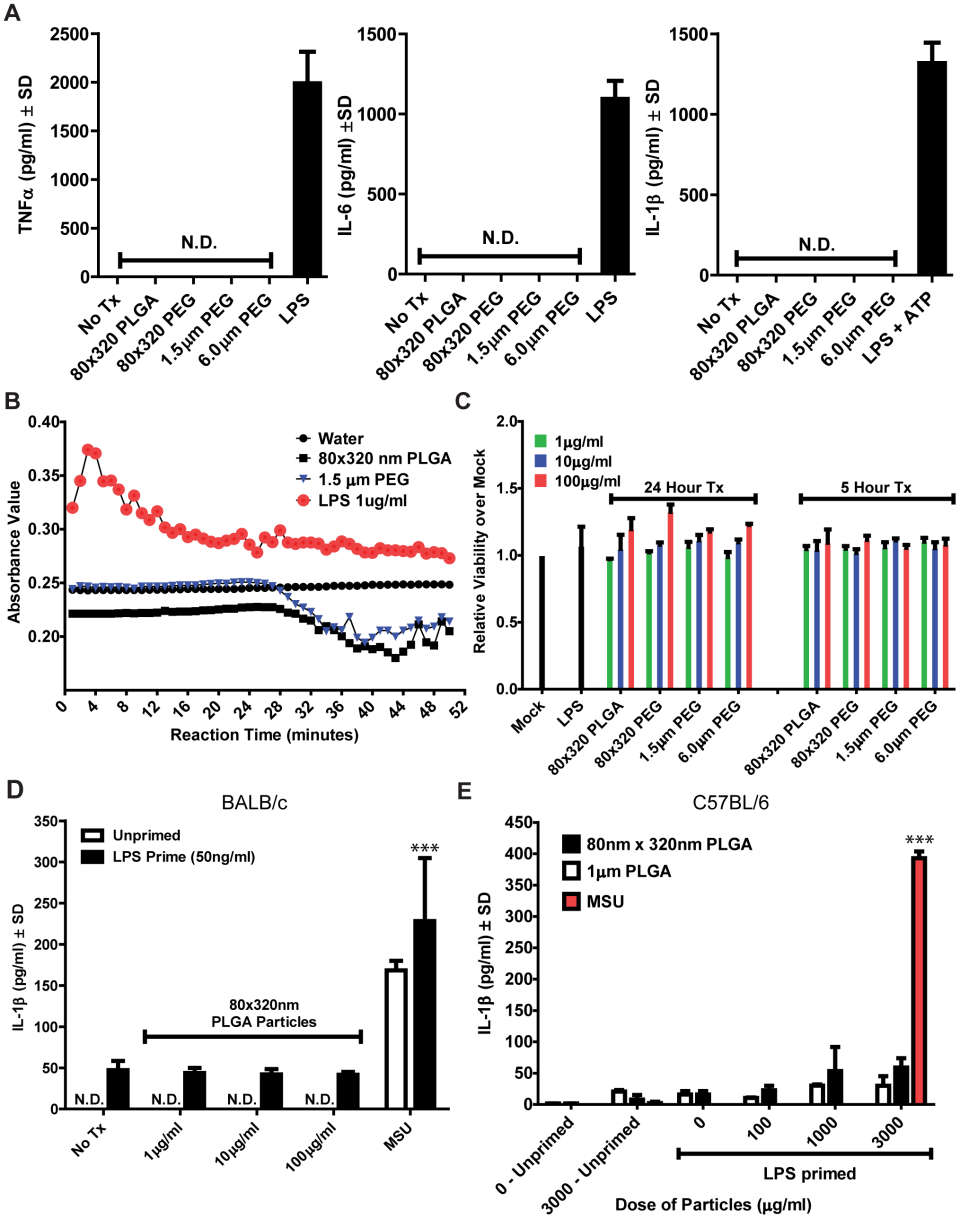
PRINT particles do not cause inflammation in bone marrow-derived macrophages from BALB/c or C57BL/6 mice. **A)** Overnight stimulation with a panel of PRINT PLGA and hydrogel particles (PEG) at 100 µg/ml does not cause TNF-α, IL-6, or IL-1β release from bone marrow-derived macrophages from C57BL/6 mice as measured by ELISA. **B)** Both PLGA and hydrogel PRINT particles (PEG) tested negative for endotoxin contamination using a Limulus amebocyte lysate assay. **C)** PRINT particles are not cytotoxic in bone-marrow derived macrophages as determined by lactate dehydrogenase (LDH) release. **D)** 80×320 nm PLGA particles do not synergize with LPS to induce inflammasome activation as measured by IL-1β ELISA in BALB/c bone-marrow derived macrophages. **E)** Neither 80×320 nm nor 1 µm PLGA particles synergize with LPS to induce inflammasome activation as measured by IL-1β ELISA in C57BL/6 bone-marrow derived macrophages. MSU was dosed at 300 µg/ml. *** = p<0.001. Experiments were performed in triplicate. Data shown are representative of at least three independent experiments.

We initially tested the inflammatory potential of PRINT particles in an *in vitro* cell culture system with bone marrow-derived macrophages from C57BL/6 mice. We used a panel of PRINT particles that differed in composition and size (Figure 1). After either a 5 hour or 24 hour incubation with a panel of PRINT particles comprised of either PLGA or PEG derivatives, we saw no detectable levels of any tested pro-inflammatory cytokine (TNF-α, IL-6, IL-1β) across a range of doses (1–100 µg/ml) (Figure 2A and data not shown). Lipopolysaccharide (LPS), a cell wall component of gram-negative bacteria, was used as a positive control for inflammation induction. The lack of cytokine induction was in line with data from endotoxin assays indicating our fabrication process was endotoxin-free (Figure 2B). In addition, across all doses of particles tested, we did not observe any particle-induce cytotoxicity as measured by LDH assay (Figure 2C).

Given the recent discovery of the inflammasome as a mediator of the innate immune response to particulate challenge, we also sought to address whether PRINT-fabricated PLGA particles could cause inflammasome activation [35], [39], [40]. The inflammasome is a multi-protein complex that is formed in response to variety of environmental stimuli, including asbestos, silica and monosodium urate crystals (MSU), that results in the activation of caspase-1 and subsequent maturation and secretion of the pro-inflammatory cytokines IL-1β and IL-18 [41]. As our initial results did not indicate any particle induction of IL-1β (Figure 2A), we next assessed whether priming macrophages with LPS would cause particles to induce inflammasome activation. LPS priming is thought to provide signal 1 to inflammasome formation by upregulating the protein levels of pro-IL-1β and NLRP3, a main component of the inflammasome complex [42], [43]. As assessed by IL-1β release, we did not see PLGA particle-induced activation of the inflammasome in the presence or absence of LPS-priming when tested in either BALB/c (Figure 2D) or C57BL/6 macrophages (Figure 2E). Importantly, we tested particles across a range of doses (100 ng–3000 µg/ml) and sizes (80×320 nm and 1 µm cylinders). These results suggest that PLGA particles across the nano and micron range do not synergize with TLR ligands (i.e., LPS) to induce inflammasome activation *in vitro* and lend further credence to the use of PLGA particles for *in vivo* applications.

### PLGA particles do not induce lung inflammation

**Figure 3:**
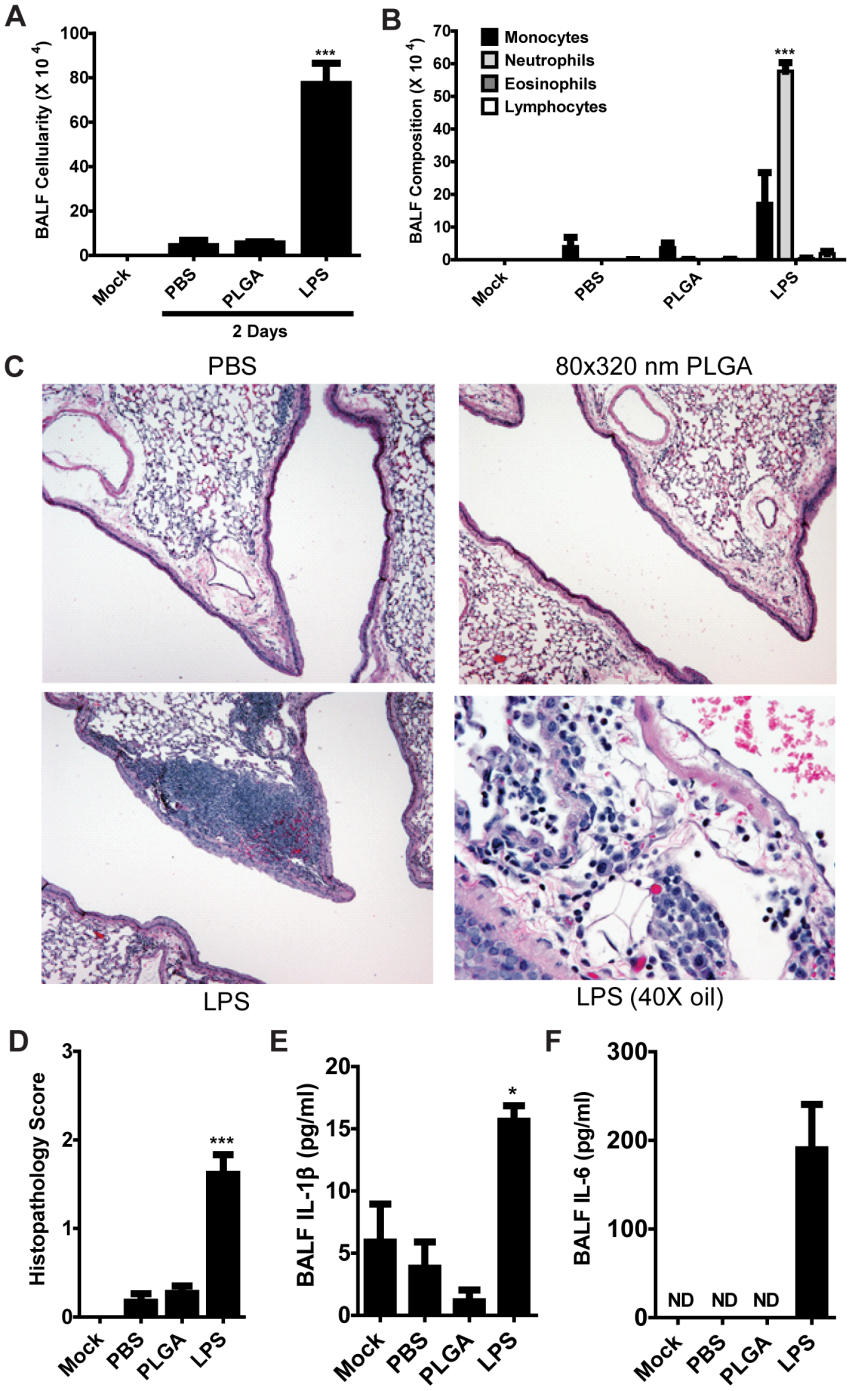
80×320 nm PLGA particles do not cause lung inflammation in mice. Mice were challenged with either 50 µg of 80×320 nm PLGA particles or 20 µg LPS i.t. and airway inflammation was assessed 48 hours post-challenge. **A)** Total cellularity of bronchoalveolar lavage fluid (BALF) in treated C57BL/6 mice is no different after 48 hours than PBS-treated mice and is significantly less than the inflammatory cell recruitment seen in LPS-treated mice. **B)** PLGA particle treatment does not induce any appreciable immune cell recruitment to the lungs of mice, as opposed to the heightened levels of monocytes and neutrophils seen in the lungs of LPS-treated mice. **C)** Histopathology revealed no significant differences in lung architecture between PBS- and 80×320 nm PLGA particle-treated mice. This is in stark contrast to the airway occlusion and significant innate immune cell recruitment seen in LPS-treated mice. **D)** Histopathology scoring confirmed that no significant differences were seen between the lungs of PBS and PLGA particle treated mice. **E–F)** The increased lung levels of pro-inflammatory IL-1β and IL-6 seen in LPS-treated mice is not found in PLGA-treated mice. PBS, n = 3; 80×320 nm PLGA particle-treated, n = 5; LPS-treated, n = 3. ND  =  Not Detected. * = p<0.05, *** = p<0.001. Experiments were performed using 3–5 mice per group. Data shown are representative of at least two independent experiments.

Bolstered by our *in vitro* findings, we next were interested in whether PLGA particles could be delivered to the lungs of mice without causing overt signs of immune activation as hallmarked by inflammation. The lung was chosen as a highly sensitive mucosal organ with clearly defined markers of inflammation that is the sight of numerous therapeutically relevant diseases, from allergies and asthma to chronic obstructive pulmonary disorder (COPD) and respiratory infections by microbial pathogens such as tuberculosis and influenza [23], [44]. As such, therapeutic modulation of lung biology is a highly desired clinical goal with relevance to the vast majority of the human population. We used intra-tracheal (i.t.) delivery to determine whether 80×320 nm PLGA particles (50 µg) caused inflammation in the lungs, with PBS (50 µl) and LPS (20 µg) used as negative and positive controls, respectively. Forty eighthours after i.t. installation, mice (n = 5 per group) were harvested and lung inflammation was assessed via field standards used in respiratory infection models [26], [27]. Broncheoalveolar lavage fluid (BALF) cellularity indicated no recruitment of immune cells to the lungs after particle treatment, as cell numbers were no different than the PBS control (Figure 3A). LPS-treated mice revealed a robust accumulation of leukocytes as is expected during inflammatory responses. Assessing the composition of leukocyte populations in the BALF revealed no significant recruitment of immune cells to the lungs of particle-treated mice. Conversely, LPS-treated mice had high levels of both monocytes and neutrophils, key mediators of the innate immune system's inflammatory response (Figure 3B).

While BALF cellularity is widely used as a marker of lung inflammation, lung histopathology enables a deeper understanding of inflammatory effects on the lung parenchyma. We examined representative sections of histopathology slides of the main bronchi of the left lobe to further delineate leukocyte infiltration around lung vasculature, parenchyma and the large and small airways (Figure 3C). Whereas LPS treatment caused a clear accumulation of leukocytes throughout the lung, treatment with 80×320 nm PLGA particles showed no difference as compared to PBS controls (Figure 3D). To further verify the non-inflammatory nature of these particles, pro-inflammatory cytokine levels were assessed in the BALF and serum of treated mice. No significant release of IL-1β (Figure 3E) or IL-6 (Figure 3F) was seen in the BALF. Serum measurements for these same cytokines and TNF-α were undetectable (data not shown). In total, these results are in agreement with our *in vitro* findings and suggest that 80×320 nm PLGA particles can be delivered to the lungs without causing innate immune activation and inflammation.

### PEG particles stably remain within the lungs for 7 days without causing lung inflammation

**Figure 4:**
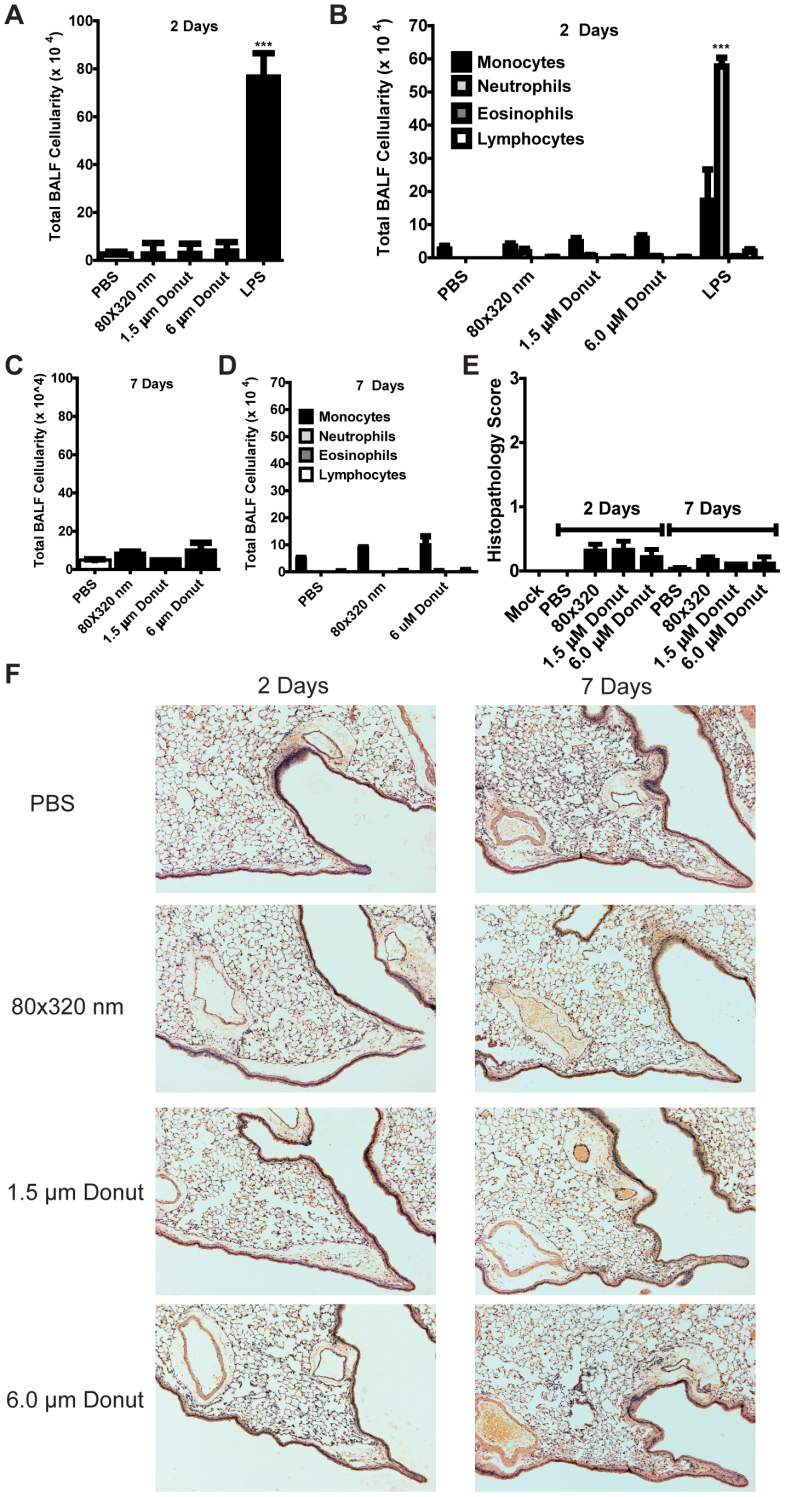
Hydrogel particles do not cause lung inflammation in mice. Mice were challenged with 50 µg of hydrogel particles (80×320 nm, 1.5 µm, or 6.0 µm donuts) i.t. and airway inflammation was assessed 48 hours and 7 days post-challenge. **A)** BALF analysis indicated no increased cellularity 48 hours after hydrogel particle treatment, whereas a significant cellular influx was seen in LPS-treated controls. **B)** At 48 hours, BALF cellular composition does not show any significant trend for immune cell recruitment in hydrogel particle-treated mice. **C–D)** BALF cellularity and composition was not significantly augmented seven days after hydrogel particle treatment. **E)** Histopathology analysis revealed no significant differences in lung architecture between PBS- and hydrogel particle-treated mice at either 2 or 7 days post-treatment. **F)** Histopathology scoring confirmed that no significant differences were seen between the lungs of PBS and hydrogel particle treated mice at any time points. *** = p<0.001. Experiments were performed using 2–5 mice per group. Data shown are representative of at least two independent experiments.

To broaden the implication of our *in vivo* findings, we fabricated a series of particles using PEG polymers and their derivatives (hydrogels) that incorporated fluorescent dyes which enabled us to track them *in vivo* over time after lung instillation. The hydrogel particles ranged in size from 80×320 nm to 1.5 µm and 6 µm as characterized in Figure 1. *In vitro* experiments indicated they did not elicit inflammatory cytokines or cell death from bone marrow-derived macrophages (Figure 2). Using the same experimental approach as outlined above, we instilled 50 µg of particles i.t. into C57BL/6 mice and assessed lung inflammation at two time points, 48 hours and 7 days post-particle instillation. As shown in Figure 4A, total BALF cellularity does not increase in the presence of hydrogel particles as compared to PBS at 48 hours, which is in marked contrast to LPS-induced cell recruitment to the lungs. Breaking down the BALF cell types revealed a similar number of monocytes in the lungs PBS and particle-treated mice, whereas LPS-treatment induced a marked influx of both monocytes and neutrophils. At 7 days post-particle treatment, there was no significant increase in the total BALF cellularity or composition in mice treated with any hydrogel particles (Figure 4C and 4D). Histopathology analyses indicated neither lung architecture disruption nor leukocyte infiltration into the lungs or airways of particle-treated mice as compared to PBS controls at either the 48 hour or 7 day time point (Figure 4E and 4F).

**Figure 5:**
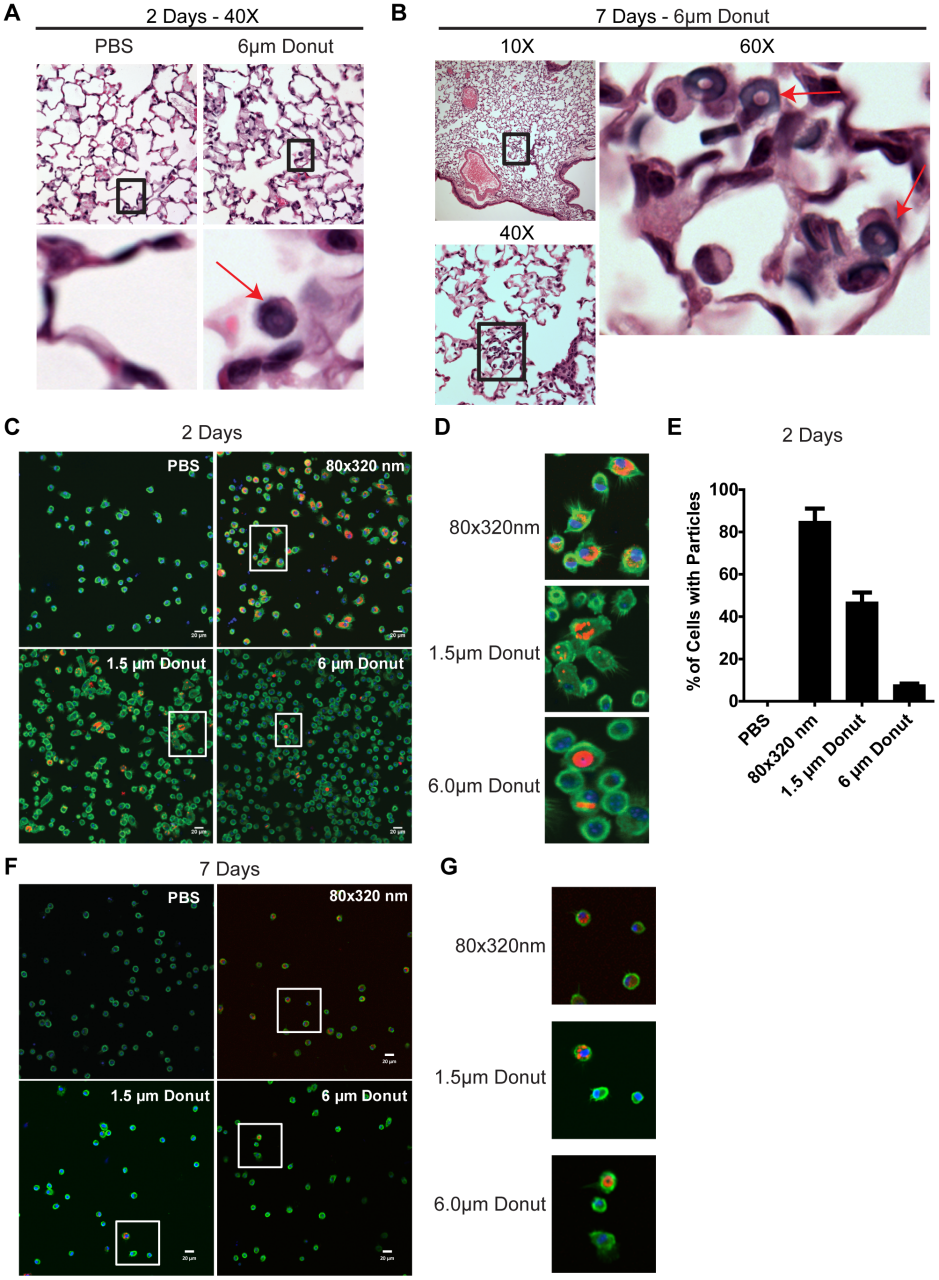
Hydrogel particles remain in the lungs for multiple days without overt signs of inflammation. **A)** 6 µm hydrogel particles (denoted by red arrows) are visible in the alveolar spaces 2 days after intratracheal installation. Lower insets are a magnified view of black bounding box. PBS treated mice are shown as control. **B)** Multiple 6 µm hydrogel particles (denoted by black bounding box and red arrows) are visible in the alveolar spaces 7 days after intratracheal installation. **C)** Two days after treatment with hydrogel particles, BALF cells were stained and visualized for particle uptake via epifluorescence microscopy. Particles (Dylight 650, red); nuclei (DAPI, blue); F-actin (Phalloidin 488, green). **D)** Magnified views of BALF cells taking up hydrogel particles as denoted by white bounding boxes in Figure C. **E)** Quantification of particle uptake indicates smaller particles are more readily taken up in BALF cells than larger particles. **F)** All types of hydrogel particles can still be seen in BALF cells seven days after treatment, though there is a marked decrease in the number of particles present as compared to the 2 day time point. **G)** Magnified views of BALF cells taking up hydrogel particles 7 days after treatment as denoted by white bounding boxes in Figure C. Scale bar is 20 µm. Data shown are representative of at least two independent experiments.

Remarkably, and despite the absence of overt signs of inflammation, 6 µm particles with their hallmark donut appearance could be viewed within the lung spaces of multiple mice by H&E staining 2 and 7 days post-challenge (Figure 5A–B). The lack of immune cell recruitment or disruption of tissue architecture around these particles may suggest an immunologically inert deposition of particles within the alveolar spaces. Such a depot may provide sustained localized delivery of therapeutically attractive molecules. Because the 80×320 nm and 1.5 µm particles were too small to see in lung histology samples, we also performed immunofluorescence imaging on BALF samples to determine whether lung-localized cells took up particles. As shown in Figure 5C, BALF cells contained hydrogel particles of all sizes at 48 hrs (magnified view in Figure 5D). We also noted that the percentage of cells with particles decreases as particles size increases (Figure 5E). Whether this is due to the quantitatively higher number of 80×320 nm particles at the same dose weight of larger particles, the relatively easier ability for a cell to take up smaller particles as compared to larger ones, or an as yet unidentified size-dependent biological effect remains unanswered. We were also interested to find that BALF cells 7 days after particle instillation show particle uptake, albeit to a lesser extent than the 48 hour time point (Figure 5F and magnified view in Figure 5G). Finally, we quantified the levels of pro-inflammatory cytokines released into the BALF and serum of PEG particle-treated mice. At both the 48 hour and 7 day time points we were unable to detect IL-1β, IL-6, or TNF-α for any particle treatment (data not shown). In total, these data highlight the ability of PRINT particles to remain localized to the lung for long periods of time in an immunologically inert manner.

## Discussion

Given the diverse therapeutic potential of nano- and microscale particles, this study sought to define whether particles composed of either PLGA or PEG-derivatives induced inflammatory responses in an *in vitro* and *in vivo* setting. By making use of the highly controlled PRINT fabrication method, we were also able to determine whether particle size affected any ensuing innate immune responses. Our findings reveal that PRINT particles do not cause any obvious activation of the innate immune response in murine macrophages or the murine lung and maintain long term (7 day) immunologic stability in the lungs of mice.

The wide array of polymers and particle fabrication techniques used in nanomedicine studies makes it difficult to reach definitive conclusions regarding particle effects on innate immune functions. We initially used particles fabricated from PLGA as this is a commonly used polymer with attractive clinical potential given its F.D.A approval. There is some discrepancy in the literature as to whether PLGA particles are inflammatory *in situ*. Some groups suggest PLGA particles are inflammatory *in vitro* and *in vivo*, whereas others have not found this to be the case [39], [45]–[48]. Our study reveals that PLGA particles of nano and micron range fabricated by PRINT technology do not synergize with a TLR ligand to cause inflammasome activation nor inflammation in general, and that *in vivo* delivery does not trigger an inflammatory reaction, contrary to a previous report [39]. The discrepancy between these findings may be due to differences in particle fabrication or experimental settings [49]. However, given the long clinical history of PLGA and the broad literature reporting PLGA particle uses for biomedical applications, it seems unlikely that particles derived from PLGA would trigger potent inflammatory responses, yet this confusion is precisely why more research must be carried out to ensure such unwanted side-effects are avoided as fabrication methods or material sourcing may impact immune responses significantly [50]–[52].

In addition, our studies using PEG particles enabled us to broaden our understanding of innate immune activation by particles comprised of a polymer composition that enables wide-ranging chemical modifications for enhanced functionality, such as cell targeting, pH-specific cargo release and siRNA incorporation as previously reported by our group and others [19], [20], [53], [54]. Interestingly, although these PEG particles are not considered biodegradable, they did not induce lung inflammation as seen with other non-degradable particles such as those comprised of polystyrene [46]. This suggests our PEG polymer composition may also be an attractive alternative from an environmental toxicology perspective in applications currently employing polystyrene particles.

The issue of innate immune activation by particles is of central relevance to the translational application of nanotechnology. While particulate vaccines against some pathogens and cancers will likely be designed to trigger localized inflammation as part of the general innate immune activation required for robust adaptive immune responses, most other biomedical applications for nano- and microparticles will benefit by avoiding such responses. Additionally, a strong immune response might lead to the undesirable outcome of rapid particle clearance as well as hypersensitivity responses. Drug delivery, diagnostic imaging and physiological bio-mimicry are examples of nanoengineering applications that may be impeded by innate immune activation. Importantly, many advances in immune modulation made available through rationally designed nano- and microscale particles such as tolerance induction in the setting of autoimmunity or organ transplantation, direct targeting of immune cell subsets and immune-skewing of pathological microenvironments such as tumors or sites of chronic inflammation, require that particles be designed initially from an inert immunological state [55]–[61]. To wit, if particles alone trigger inflammatory responses that skew towards any type of adaptive response (e.g., Th1, Th2) then many of these therapeutic goals will not be achieved. For these reasons, we feel it is of utmost importance that baseline innate immune responses to particles be assessed as part of field standards [9].

Our *in vivo* studies reveal that particle size augments uptake into innate immune cells of the lungs, with larger particles taken up less than smaller particles. This finding suggests a duality in design considerations depending on therapeutic application. For example, drug delivery to the lungs to ameliorate asthma would likely be best served by larger particles that can release their cargo to extracellular spaces. Conversely, if trying to deliver a respiratory vaccine, smaller particles that are more readily taken up by antigen presenting cells and traffic to lymph nodes would be more appropriate. Our finding that particles of all tested sizes remain in the lungs up to 7 days post instillation also suggest the ability to provide sustained localized delivery of therapeutically attractive molecules via particulate formulations. This is far different than the rapid clearance seen for smaller particles (<50 nm diameter) and reflects the importance of particle design parameters when considering therapeutic interventions [62].

The lung serves as an attractive route for therapeutic delivery due to its ease of access and its large absorptive surface area. There are several important particle characteristics that need to be considered for effective pulmonary delivery such as size, shape, surface charge, toxicity, and potential inflammatory effects. Inhaled particles with mass median aerodynamic diameters (MMAD) larger than 5 µm tend to be deposited in the upper conducting airway while particles with MMAD between 1–5 µm deposit in the lower respiratory airways [63]. Using PRINT, we have the ability to design particles with aerodynamically relevant deposition characteristics while having distinct non-spherical geometries enabling different deposition profiles in the lung [64], [65]. The investigation of the safety profile and inflammatory response of these inhaled polymeric particles is important to support their use as drug delivery vehicles as highlighted in this study.

Having identified particles across the nano- and microscale that do not trigger inflammatory responses in mice while remaining in the lungs, we plan to next use these particles as delivery devices for a range of biologically relevant molecules, including siRNAs, anti-inflammatory agents, and immune-skewing compounds. It should also be noted that mucoadhesive components may be incorporated within the PRINT particle system to enhance adsorption in the mucosal region which may enable differential deposition and enhanced temporal localization to the lungs. These studies will test the hypothesis that targeted modulation of lung immunology via nanoengineering may enable a new class of therapeutics for lung disorders that avoid systemic side-effects while also reducing administration doses. While we have tested a 50 µg dose of inert particles in this manuscript, PRINT enables a high weight percent loading of bioactive molecules and thus local and sustained pulmonary delivery may show therapeutic efficacy at low particle doses [19]–[22], [66]–[68].

Importantly, we will move studies into human cells to provide much needed data regarding the immunological response to nanomaterials in our own species. Using the design control inherent to PRINT technology, we will also be able to systematically address the role of particle size and shape during delivery of bioactive molecules. Such results may be crucial to advancing next-generation respiratory vaccines and treatments for asthma, allergies and chronic disorders of lung function.
